# Effect of Austenitization Conditions on the Transformation Behavior of Low Carbon Steel Containing Ti–Ca Oxide Particles

**DOI:** 10.3390/ma12071070

**Published:** 2019-04-01

**Authors:** Chao Wang, Xin Wang, Jian Kang, Guo Yuan, Guodong Wang

**Affiliations:** The State Key Laboratory of Rolling and Automation, Northeastern University, Shenyang 110819, China; chao_neu@163.com (C.W.); xinwang_research@163.com (X.W.); kang21jian@sina.com (J.K.); wanggdneu@163.com (G.W.)

**Keywords:** low carbon steel, austenitization, Ti–Ca oxide, acicular ferrite, Mn-depleted zone, boron segregation

## Abstract

Inclusion-induced acicular ferrite (AF) nucleation has been used for microstructure refinement in steel. Austenitization conditions have a significant influence on AF nucleation ability. In this paper, the effects of austenitization temperature and holding time on the transformation behaviors of low carbon steel containing Ti–Ca oxide particles were studied. A thermal treatment experiment, high temperature in situ observation, and calculation of Mn diffusion were carried out. The results indicate that small austenite grain size under low austenitizing temperature promoted grain boundary reaction products. With an increase in austenitizing temperature, the nucleation sites transferred to intragranular particles and AF transformation was improved. The inclusion particles in the Ti–Ca deoxidized steel were featured by an oxide core rich in Ti and a lesser amount of Ca and with MnS precipitation on the local surface, which showed a strong ability to promote AF nucleation. At a low austenitizing temperature, Mn diffusion was limited and the development of Mn-depleted zones (MDZs) around inclusions was not sufficient. The higher diffusion capacity of Mn at a high austenitizing temperature promoted the formation of MDZs to a larger degree and increased the AF nucleation ability. Boron segregation at large-sized austenite grain boundaries played an important role in AF transformation. Austenite grain size, Mn-depleted zone development, and boron segregation at grain boundaries were the decisive factors influencing the transformation behaviors under different austenitization conditions for the test steel.

## 1. Introduction

An intragranular acicular ferrite (AF)-type microstructure is characterized by fine grain size and high-angled grain boundaries, which contribute to improving steel’s comprehensive properties of strength and toughness [1,2]. Inclusion-induced AF transformation has been extensively researched and applied to various steel products. The most significant application is toughness improvement of the steel welding heat-affected zone (HAZ) and weld metal (WM) [3,4]. Moreover, it is also used to enhance the performance of steel base metal including low carbon non-quenched and tempered steel, high-strength low-alloy (HSLA) steel, and so on [5,6]. The effect of inclusion on AF formation in steel has been summarized in some review articles [7,8,9]. The formation of AF in steel is influenced by various factors, such as inclusion size and composition, prior austenite grain size, cooling rate, and so on.

Ti-oxide particles are considered especially effective for AF nucleation. However, TiO_x_ inclusions formed during steel melt deoxidation easily coagulate and deteriorate the steel’s properties [10]. Therefore, complex deoxidation is usually adopted to obtain fine oxide particle distribution, such as Ti–Mg deoxidation, Ti–Zr deoxidation, and Ti–Al–Mg/Zr deoxidation [11,12,13,14]. Recently, growing concerns have been focused on Ti–Ca complex treatment. Modification behavior of non-metallic inclusions during the Ca treatment process has been explored [15]. Zheng et al. reported that Ca treatment received better effect in Al–Ti deoxidized steel [16]. Zhang et al. found that Ca could also modify solid Al–Ti inclusions to spherical particles [17].

It is widely accepted that oxide particles tend to be stable at high temperatures and induce AF nucleation during cooling. In welding HAZ, the effect of oxide particles is always evaluated under conditions of a high peak temperature around 1400 °C and a short holding period between several and a few tens of seconds [18]. However, with regard to hot rolled or forged steels, relatively low temperatures and long austenitization times are concerned. The effectiveness of oxide inducing AF nucleation and the relevant Mn-depleted zone (MDZ) mechanism remains to be further verified. Results of Wen et al. showed that AF could well nucleate with an austenitizing temperature at 1200 °C in Ce deoxidized steel [19]. Effects of austenitizing temperature and holding time on microstructure transformation at various cooling rates in Ti–Ca deoxidized steel have not been researched. The present study is aimed to clarify the influence of austenitization parameters on the effect of Ti–Ca oxide particles on microstructure evolution behaviors in low carbon steel, which will be conducive to its application in hot rolled or forged steel products.

## 2. Materials and Methods

Experimental low carbon steel was melted in a 50 kg vacuum induction melting furnace and cast into ingots. The basic chemical composition was 0.07C–0.06Si–1.5Mn–0.2Cr–0.001B–0.01Ti–0.001Ca–0.0025N–0.004O (wt.%). The test steel was deoxidized with Ti–Ca compound deoxidant. The deoxidization process was deliberately controlled to avoid the detrimental effect of coarse inclusions and to get a uniform distribution of fine inclusions. Boron was added to improve the inclusion-induced nucleation of intragranular acicular ferrite.

**Figure 1:**
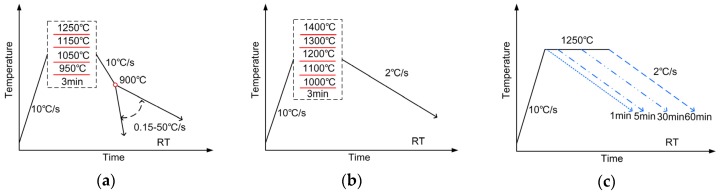
Schematic diagrams of thermal treatment processes for (**a**) continuous cooling transformation, (**b**) in situ observation and (**c**) austenitization holding.

The effect of austenitizing temperature on continuous cooling transformed microstructures at different cooling rates was investigated adopting the scheme shown in Figure 1a. The experiment was carried out on Formastor-FⅡ full-automatic transformation equipment using cylinder samples of dimensions Φ3 mm × 10 mm. Vickers hardness of treated samples was tested by a KB hardness testing machine (KB Prüftechnik, Hochdorf-Assenheim, Germany). In order to clarify the transformation behaviors in detail, in situ observation by high-temperature laser scanning confocal microscopy (LSCM) was carried out, referring to the thermal cycle process in Figure 1b. The samples were machined into disks with a diameter of 7 mm and thickness 3.5 mm, mirror polished, and set into an alumina crucible on a laser scanning confocal microscope (LSM 510, Carl Zeiss, Jena, Germany). The austenite coarsening and ferrite formation behaviors were directly observed. The effect of austenitization holding time was also evaluated through experiment as indicated in Figure 1c. Steel samples were heated at 1250 °C and held for different times between 1 and 60 min and then continuously cooled at 2 °C/s.

Microstructure characterization was carried out using an Olympus BX51 optical microscope (OM, Olympus Corporation, Tokyo, Japan) and a Zeiss Ultra-55 scanning electron microscope (SEM, Carl Zeiss, Jena, Germany). Inclusions in steel were analyzed by a JXA-8530F electron probe microanalyzer (EPMA, JEOL Ltd., Tokyo, Japan) equipped with an energy dispersive spectroscopy (EDS) system. Meanwhile, Mn diffusion in austenite at different temperatures was calculated by Thermo-Calc DICTRA dynamics software (V2017b, Thermo-Calc Software AB, Solna, Sweden) with MOBFE4 database.

## 3. Results

### 3.1. Microstructures Formed at Various Cooling Rates

**Figure 2:**
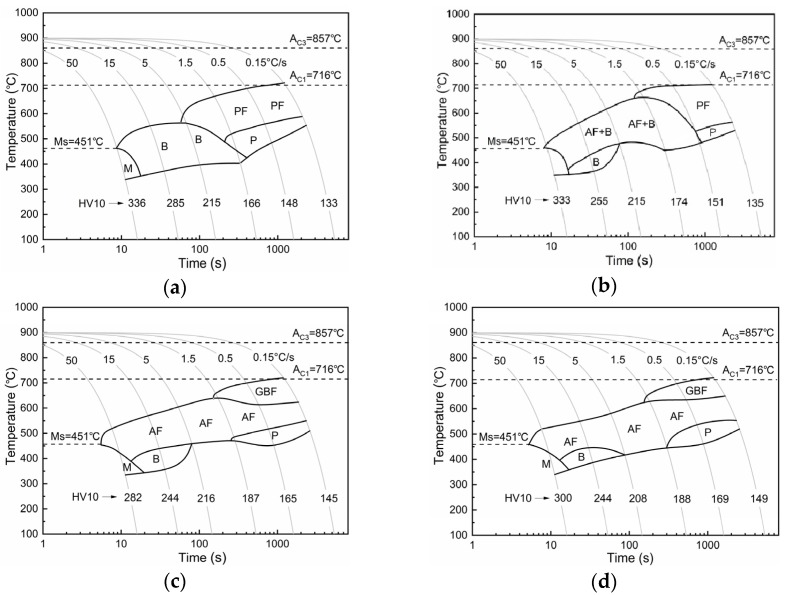
Continuous cooling transformation (CCT) diagrams of test steel austenitized at (**a**) 950 °C, (**b**) 1050 °C, (**c**) 1150 °C, and (**d**) 1250 °C.

Combining dilatometry and microstructural characterization, continuous cooling transformation (CCT) diagrams of test steels with different austenitization temperatures are presented in Figure 2. With a low austenitization temperature at 950 °C (Figure 2a), the normal ferrite, bainite (B), and martensite (M) microstructures were formed respectively with the increase in cooling rate. Ferrite grains forming at low cooling rates were polygonal ferrite (PF), and pearlite (P) then formed between the PF grains. When the austenitization temperature increased to 1050 °C (Figure 2b), acicular ferrite (AF) appeared together with bainite, while the PF transformation region shrunk. With an austenitization temperature above 1150 °C (Figure 2c,d), the formation of AF occurred at all experimental cooling rates: 0.15–50 °C/s. At low cooling rates, grain boundary ferrite (GBF) formed before AF transformation and pearlite formed between AF laths. At high cooling rates, complex microstructures of AF and B/M were obtained. Full acicular ferrite structure was formed at medium cooling rates around 5 °C/s.

**Figure 3:**
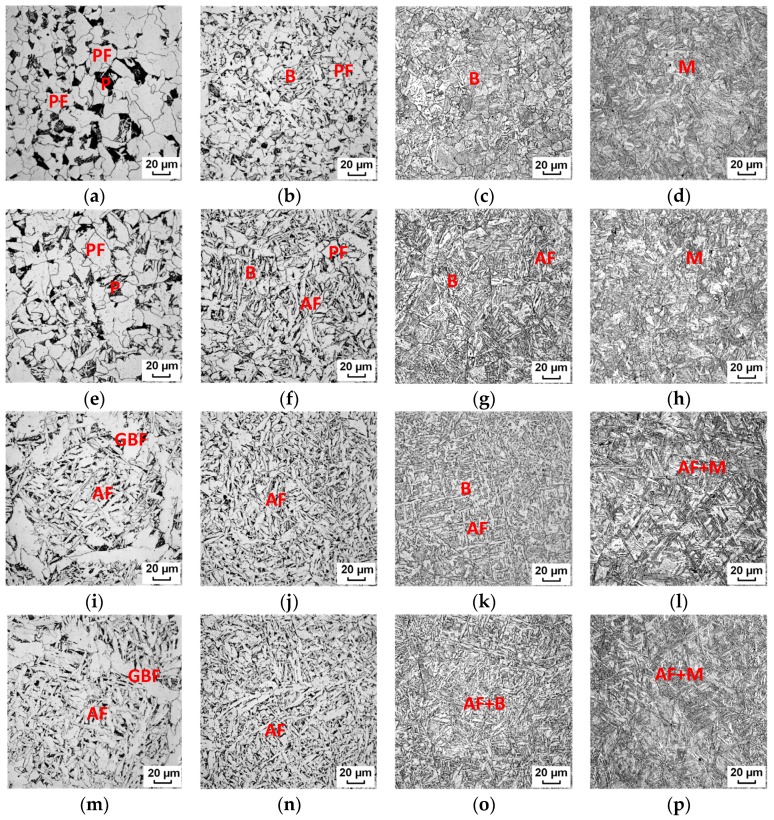
Microstructures formed at cooling rates of (**a**,**e1**,**i**,**m**) 0.5 °C/s, (**b**,**f**,**j**,**n**) 1.5 °C/s, (**c**,**g**,**k**,**o**) 15 °C/s, and (**d**,**h**,**l**,**p**) 50 °C/s with austenitization temperatures of (**a**–**d**) 950 °C, (**e**–**h**) 1050 °C, (**i**–**l**) 1150 °C, and (**m**–**p**) 1250 °C. PF: Polygonal ferrite; AF: Acicular ferrite; GBF: Grain boundary ferrite; P: Pearlite; B: Bainite; M: Martensite.

The corresponding microstructures are shown in Figure 3. With a low austenitization temperature of 950 °C, large-sized polygonal ferrite (PF) and pearlite were formed at a cooling rate of 0.5 °C/s. Ferrite grains became finer at 1.5 °C/s and bainite or martensite formed at higher cooling rates. With an austenitization temperature above 1050 °C, the microstructure morphology changed greatly. Acicular ferrite began to form evidently and its volume fraction increased with an increase in austenitization temperature. At a lower cooling rate of 0.5 °C/s, grain boundary allotriomorphic ferrite (GPF) still accounted for a considerable volume fraction. At 1.5 °C/s, almost a full AF structure was obtained with an austenitization temperature above 1150 °C. When the cooling rate rose to 15–50 °C/s, complex microstructures of AF–bainite or AF–martensite were formed. AF plates formed firstly at higher temperatures during cooling, and then bainite or martensite packets formed between AF plates.

The result indicates that under a low austenitizing temperature of 950 °C, the inclusions did not have much influence on microstructure transformation. On the one hand, austenite grain size was small, and the grain boundary area increased, which promoted grain boundary reaction products such as GBF and bainite [20]. On the other hand, at lower austenitizing temperatures, the formation of the MDZ around inclusions was not sufficient, which would reduce the ability to induce AF nucleation. Higher austenitizing temperatures would promote wider MDZ development and AF formation [21].

### 3.2. Transformation Behaviors by In Situ Observation

**Figure 4:**
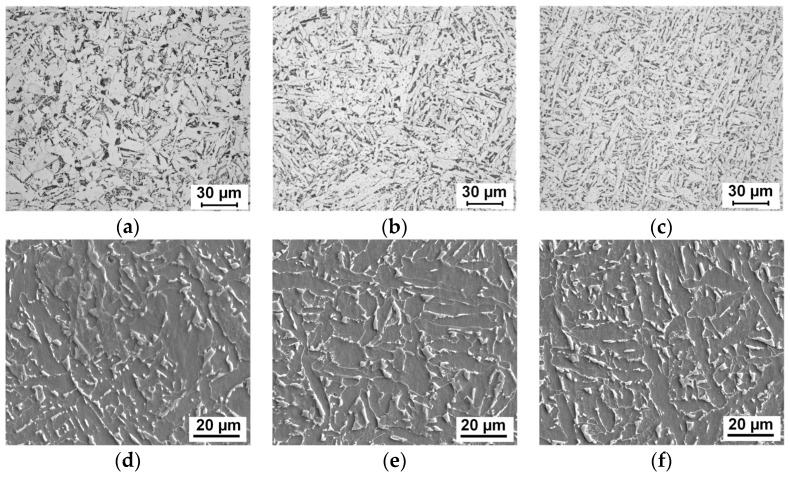
Optical (**a**–**c**) and SEM (**d**–**f**) microstructures formed with austenitizing temperatures of (**a**,**d**) 1000 °C, (**b**,**e**) 1200 °C, and (**c**,**f**) 1400 °C.

Test steel samples were austenitized at 1000–1400 °C for 3 min and cooled at 2 °C/s on LSCM. The final samples were cut in half and the inside microstructures were observed by OM and SEM, as shown in Figure 4. At the same cooling rate of 2 °C/s, with an increase in austenitizing temperature, the microstructure morphology exhibited a polygonal ferrite→bainite→acicular ferrite variation trend. The nucleation sites transferred from grain boundaries to intragranular inclusions with an increase in the austenitizing temperature. It is generally accepted that larger austenite grains promote acicular ferrite nucleation. Lee et al. found that the relationship between the acicular ferrite fraction and austenite grain size followed a C-type curve [22]. On the other hand, under high-temperature conditions, the development of Mn depletion zones around oxide particles would also promote acicular ferrite formation significantly. It will be further discussed in Section 4.

**Figure 5:**
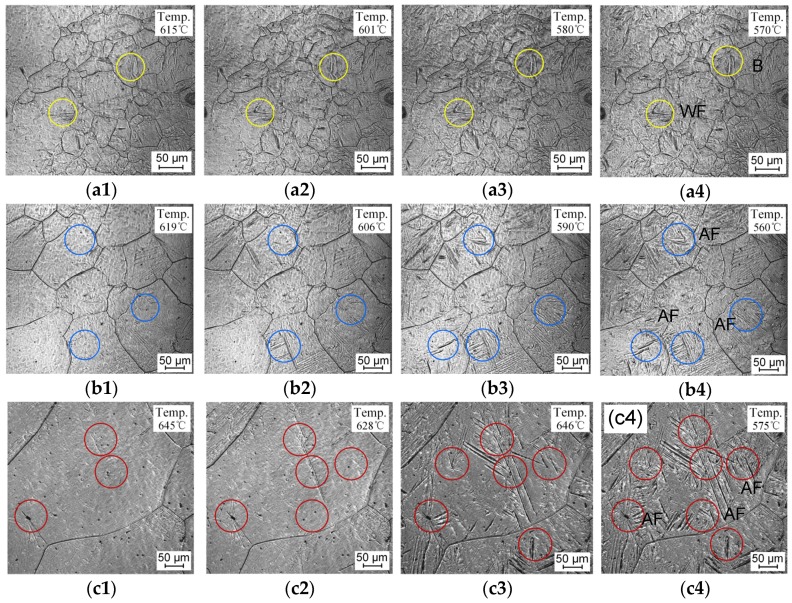
In situ micrographs by laser scanning confocal microscopy (LSCM) during cooling with austenitizing temperatures of (**a1**–**a4**) 1000 °C, (**b1**–**b4**) 1200 °C, and (**c1**–**c4**) 1400 °C.

**Figure 6:**
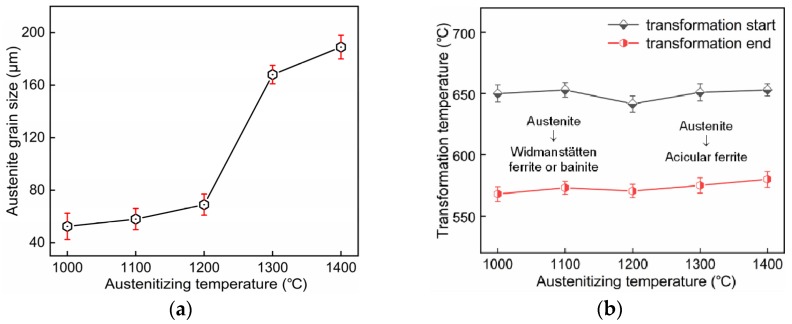
Effects of austenitizing temperature on (**a**) austenite grain size and (**b**) transformation temperature.

A series of in situ micrographs taken by LSCM during sample cooling is shown in Figure 5. Inclusion particles appear as small black dots in the LSCM micrographs. Ferrite transformation always occurred first at the austenite grain boundaries in all the test samples. Acicular ferrite plates were observed to nucleate on the intragranular inclusions during cooling. Besides, acicular ferrite lath could also form on the surface of the pre-existing ferrite lath. It is possible that the newly formed acicular ferrite was sympathetically nucleated on the broad face of pre-formed acicular ferrite. The average sizes of prior austenite grains after 3 min holding at peak temperatures were measured with the linear intercept method. In situ micrographs with magnifications of ×200 and ×100 were used for samples with austenitization temperatures at 1000–1200 °C and 1300–1400 °C, respectively. The result is presented in Figure 6a. It shows that the average size of the austenite grains increased from 53 μm to 189 μm when the peak temperature increased from 1000 °C to 1400 °C. Babu et al. reported that acicular ferrite nucleates at non-metallic inclusions and grows within prior austenite grains competing with the growth of Widmanstätten ferrite and bainite, which nucleate at grain boundaries [23]. Austenitization at a relatively low temperature of 1000 °C caused a remarkable increase in grain boundary reaction products. By contrast, acicular ferrite forming intragranularly was significantly improved at higher austenitizing temperatures of 1200–1400 °C. This indicates that a high temperature contributes to inclusion-induced nucleation, which is consistent with the result in Figure 3. In addition, the austenitizing temperature also influenced the transformation temperatures during cooling, as presented in Figure 6b. The formation of acicular ferrite tended to raise the transformation temperature. For the 1400 °C austenitized sample, AF transformation was completed in the range of 660–561 °C. The effect of the austenitizing temperature on AF transformation lies not only in its influence on austenite grain size but also on the diffusion of manganese atoms, which may affect the development of Mn-depleted zone [24].

## 4. Discussion

**Figure 7:**
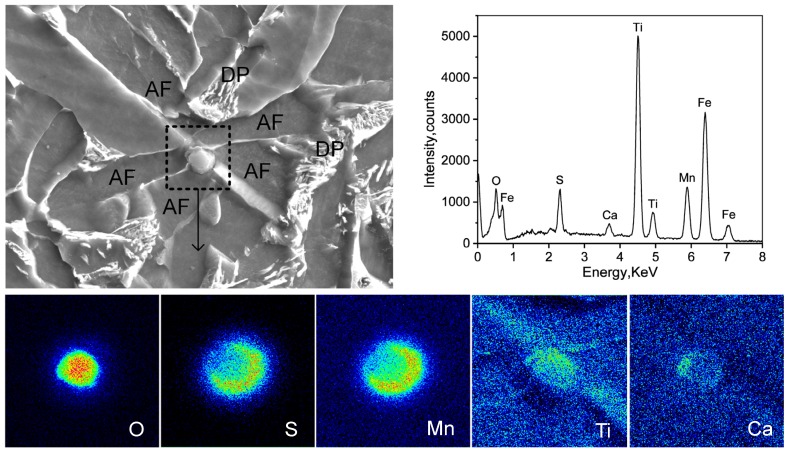
Electron probe microanalyzer (EPMA) analysis of one typical inclusion particle effective for AF nucleation in experiment steel. AF: Acicular ferrite; DP: Degenerated pearlite.

A typical inclusion particle in steel was analyzed by EPMA as shown in Figure 7. Several AF plates emanated from the surface of the inclusion. The oxide core was rich in Ti and O, as well as a lesser amount of Ca; MnS precipitated on the local surface. The Ti–Ca–O–Mn–S-type complex inclusion was predominant in the Ti–Ca deoxidized steel and was effective for AF nucleation. The development of Mn-depleted zones through both Mn absorption into Ti–oxide and MnS precipitation was the main factor promoting AF nucleation. A decrease in the Mn content around the particles will result in a reduction in austenite stability and increase the driving force for ferrite transformation. Thus, ferrite nucleation will first occur on intragranular particles leading to the formation of an interlocking AF microstructure.

**Figure 8:**
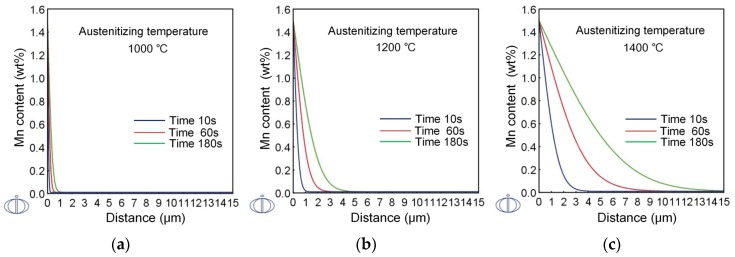
Diffusion distance of Mn in austenite at different (**a**) 1000 °C, (**b**) 1200 °C and (**c**) 1400 °C.

Inclusion-induced AF nucleation is usually considered in welding the coarse-grained heat-affected zone (CGHAZ) where the peak temperature is around 1400 °C and the holding time is several to a few tens of seconds. However, for steel base metal, the heating temperature is not so high and the holding time is longer. In this case, the formation of MDZ will be affected as well as the ability of AF nucleation. Mn diffusion behaviors in austenite at different temperatures were calculated by Thermo-Calc DICTRA dynamics software, as shown in Figure 8.

Figure 8 indicates that the diffusion capacity of Mn is promoted significantly with the increase in temperature. Mn diffusion distance within 3 min increased from 0.27 μm to 4.2 μm as the temperature rose from 1000 °C to 1200 °C. However, the diffusion distance reached up to 4.5 μm in just 10 s at 1400 °C. Improvement in the Mn diffusion capacity contributed to the development of MDZ of higher degrees and increased the AF nucleation ability [25]. At low austenitizing temperatures under 1000 °C, Mn diffusion and MDZ formation were difficult, while austenite grains were much finer. This caused the grain boundaries to act as active nucleating sites while inclusions were ineffective. With high austenitizing temperatures above 1250 °C, the coarse austenite grain size and MDZ formation together promoted AF transformation. Meanwhile, Shim et al. also reported that Ti_2_O_3_ particles do not become effective nucleation sites for acicular ferrite at low austenitization temperatures [26].

**Figure 9:**
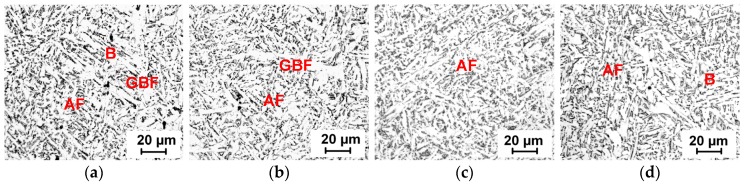
Optical microstructures formed with different holding times at 1250 °C: (**a**) 1 min, (**b**) 5 min, (**c**) 30 min, (**d**) 60 min. AF: Acicular ferrite; GBF: Grain boundary ferrite; B: Bainite.

**Figure 10:**
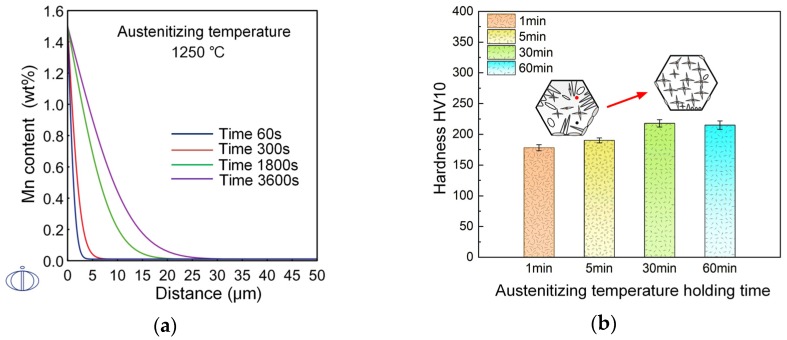
(**a**) Mn diffusion distance and (**b**) macro-hardness of steel samples with different hold times at 1250 °C.

For wrought steel products, steel billets are usually heated above 1200 °C with a long soaking time. The effect of Mn diffusion on AF transformation under this condition should be further considered. Figure 9 shows the microstructures formed with austenitization at 1250 °C for 1–60 min. For a short holding time of one minute, bainite sheaves were still observed (Figure 9a). As the austenitization holding time increased to 30 min, full acicular ferrite microstructures were formed (Figure 9c). Grain boundary ferrite was also inhibited, which existed in Figure 9a,b. For a long holding time of 60 min, bainite tended to form again, as shown in Figure 9d. The hardness of the steel samples also varied in accordance with the microstructure evolution, which is presented in Figure 10b. Austenite grains had grown sufficiently large for AF formation after heating to 1250 °C. The transformation behaviors were, thus, mainly influenced by Mn diffusion during different holding times. The calculation results of Mn diffusion at 1250 °C are presented in Figure 10a. It indicates that the holding time has an influence on Mn diffusion distance. Within one minute, Mn diffusion was not sufficient and MDZ had not developed well, such that the inclusion-induced nucleation effect decreased. As the holding time increased to 30 min, the formation of full AF microstructures indicated that MDZs around inclusions were well developed. However, after 60 min holding, acicular ferrite nucleation ability seemed to exhibit a declining tendency. It is speculated that after saturation of Mn in oxide, the extent of MDZ would decrease due to the continued diffusion of Mn atoms. Further studies and confirmation need to be carried out.

In the tested steel, 0.001 wt.% boron was added to promote acicular ferrite formation. Boron had a significant influence on the effectiveness of oxide particles under different austenitizing conditions. Segregation of boron atoms at the austenite grain boundaries could retard the austenite-to-ferrite transformation occurring at the boundaries. It was shown that ferrite side plates or bainite start temperatures were reduced by about 50 °C during continuous cooling because of boron segregation [27]. Moreover, boron does not segregate on a Ti-oxide surface due to its diffusion into the oxide particle, which contains dense cation vacancies [24]. Thus, boron would not reduce the intragranular nucleation ability. In the present study, with an increase in austenitization temperature, the austenite grain size grew coarse and the segregation degree of boron at the grain boundaries became higher. The grain boundary ferrite, ferrite side plates, or bainite transformation will be further inhibited, resulting in acicular ferrite formation to a greater extent. Therefore, in addition to MDZ development, boron segregation at the austenite grain boundaries and absorption into Ti-oxide particles is an important factor influencing the transformation behaviors under different austenitization conditions.

## 5. Conclusions

The effects of austenitization conditions on the transformation behaviors of low carbon steel-containing Ti–Ca oxide particles were studied. The main conclusions are as follows:

(1) With an increase in austenitizing temperature, the continuous cooling microstructures exhibit a polygonal ferrite→bainite→acicular ferrite variation trend. Small austenite grain size promotes grain boundary reaction products such as GBF and bainite. With an increase in austenitizing temperature, the nucleation sites transfer from grain boundaries to intragranular particles.

(2) With high austenitizing temperatures, full acicular ferrite transformation could complete between 660 °C and 561 °C during 2 °C/s cooling. The oxide particles introduced by Ti–Ca deoxidation play an important role in promoting AF transformation. The particles are featured by an oxide core rich in Ti and a lesser amount of Ca, with MnS precipitation on the local surface. These particles show a strong ability to promote AF nucleation and the Mn-depleted zone is the operative mechanism.

(3) At a low austenitizing temperature, Mn diffusion is limited and the development of an MDZ around inclusions is not sufficient, which would reduce its nucleating ability. The diffusion capacity of Mn increased significantly with an increase in austenitizing temperature and holding time, which promotes the formation of MDZs to a larger degree and increases AF nucleation ability.

(4) The influence of austenitization conditions on the transformation behavior of the test steel takes effect from three aspects, i.e., austenite grain size, Mn-depleted zone development, and boron segregation at grain boundaries.
